# Positive Youth Development Constructs: Conceptual Review and Application

**DOI:** 10.1100/2012/152923

**Published:** 2012-05-03

**Authors:** Daniel T. L. Shek, Rachel C. F. Sun, Joav Merrick

**Affiliations:** ^1^Department of Applied Social Sciences, The Hong Kong Polytechnic University, Hong Kong; ^2^Public Policy Research Institute, The Hong Kong Polytechnic University, Hong Kong; ^3^Department of Social Work, East China Normal University, Shanghai 200241, China; ^4^Kiang Wu Nursing College of Macau, Macau, China; ^5^Division of Adolescent Medicine, Department of Pediatrics, Kentucky Children's Hospital, University of Kentucky College of Medicine, Lexington, KY 40506-9983, USA; ^6^Faculty of Education, The University of Hong Kong, Hong Kong; ^7^National Institute of Child Health and Human Development, P.O. Box 1260, IL-91012 Jerusalem, Israel

In the prevention science approach, focus is put on identifying risk and protective factors in adolescent risk behavior. Based on this approach, many research and prevention programs have been generated in the past few decades. Nevertheless, overemphasis of adolescent developmental problems has been criticized as focusing too much on adolescent developmental problems and pathologies. In response to this criticism, an alternative approach highlighting the importance of positive youth development has been proposed. According to Damon [1], the field of positive youth development (PYD) focuses on each child's talents, strengths, interests, and future potential in contrast to approaches that focus on problems that youth display when they grow up, such as learning disabilities and substance abuse. Catalano et al. [2] pointed out that there are several characteristics associated with the positive youth development approach, including emphasis on integrated youth development (i.e., focusing on a range of youth developmental possibilities and problems) rather than dealing with a single youth problem, upholding the belief that “problem-free is not fully prepared,” emphasis of person-in-environment perspective, and focus on developmental models about how young people grow, learn, and change.

 There are different models on the developmental potentials and abilities of young people. For example, Benson [3] proposed 40 developmental assets that should be nurtured in adolescents. In the same vein, many researchers suggest that building cognitive, academic, social, and emotional competence is a fundamental task in adolescence [4]. According to the Collaborative for Academic, Social and Emotional Learning (CASEL), “social and emotional learning (SEL) is the process of acquiring the skills to recognize and manage emotions, develop caring and concern for others, make responsible decisions, establish positive relationships, and handle challenging situations effectively. Research has shown that SEL is fundamental to children's social and emotional development—their health, ethical development, citizenship, academic learning, and motivation to achieve. Social and emotional education is a unifying concept for organizing and coordinating school-based programming that focuses on positive youth development, health promotion, prevention of problem behaviors, and student engagement in learning” [5]. Generally speaking, several SEL attributes are commonly included in different SEL models. These include self-awareness (identifying emotions and recognizing strengths), social awareness (perspective-taking and appreciating diversity), self-management (managing emotions and goal setting), responsible decision making (analyzing situations, assuming personal responsibility, respecting others, and problem solving), and relationship skills (communication, building relationships, negotiation, and refusal). Research findings have shown that higher positive youth development predicted fewer problem behaviors, thus suggesting that positive youth development is an important protective factor of adolescent problem behavior [6, 7].

In a review of existing programs on positive youth development, Catalano et al. [8] identified 25 successful programs out of 77 programs they reviewed. In the successful programs, 15 positive youth development constructs were covered in the interventions. These constructs include the following.

1. *Promotion of bonding: *development of strong affective relationship with and commitment to people (healthy adults and positive peers) and institutions (school, community, and culture). According to interpersonal and family theories, adolescent developmental problems are regarded as outcomes of problem family and interpersonal processes.
2. *Promotion of social competence*: interpersonal skills (such as communication, assertiveness, conflict resolution, and interpersonal negotiation), ability to build up positive human relationship, and provision of opportunities to practice such skills. Deficits in social competence in young people are commonly related to adolescent developmental issues.
3. *Promotion of emotional competence*: awareness of one's own emotions, ability to understand others' emotions, ability to use the vocabulary of emotion, capacity for empathy, ability to differentiate internal subjective emotional experience from external emotional expression, and capacity for emotional management.
4. *Promotion of cognitive competence*: cognitive abilities, processes, or outcomes such as logical thinking, creative thinking, and critical thinking. Poor cognitive competence is usually a precursor of adolescent developmental problems.
5. *Promotion of behavioral competence*: ability to use nonverbal and verbal strategies to perform socially acceptable and normative behavior in social interactions and to make effective behavior choices, such as resisting peer pressure.
6. *Promotion of moral competence*: orientation to perform ethical behavior, ability to judge moral issues, as well as promoting the development of justice and altruistic behavior in adolescents. It is noteworthy that moral confusion is a common problem among contemporary young people.
7. *Development of self-efficacy*: beliefs in one's abilities and to use such abilities to attain certain goals. Research findings show that self-efficacy is positively related to adolescent developmental outcomes.
8. *Fostering prosocial norms*: clear and healthy standards, beliefs, and behavior guidelines which promote prosocial behavior such as cooperation and sharing.
9. *Cultivation of resilience*: ability of an individual for adapting to changes in a healthy way, a reintegration process for an individual to recover, or positive outcomes after experiencing adversity. It refers to adolescents' capacity against developmental changes and life stresses in order to “bounce back” from stressful life experience and achieve healthy outcomes.
10. *Cultivation of self-determination*: ability to set goals and make choices according to his/her own thinking. Regarding skills and strategies which promote self-determination, they include self-awareness of strengths and limitations, goal setting and action planning, problem solving, choice-making, and self-evaluation.
11. *Cultivation of spirituality*: promotion of the development of beliefs in a higher power, cultivation of a sense of life meaning, and values about life choices.
12. *Promotion of beliefs in the future*: hope and optimism, including valued and attainable goals, positive appraisal of one's capability and effort (a sense of confidence), and positive expectancies of the future.
13. *Development of clear and positive identity*: building of self-esteem and facilitation of exploration and commitments in self-definition.
14. *Opportunity for prosocial involvement*: events and activities that promote young people's participation in prosocial behaviors and maintenance of prosocial norms.
15. *Recognition for positive behavior*: development of systems for rewarding or recognizing participants' positive behavior such as prosocial behavior or positive changes in behavior.

These 15 positive youth development constructs have been used in a project entitled Positive Adolescent Training through Holistic Social Programmes (Project P.A.T.H.S.) which was initiated and financially supported by The Hong Kong Jockey Club Charities Trust (HK$400 million for the initial phase and HK$350 million in the extension phase). There are two tiers of programs in this project. While the Tier 1 Program is a curriculum-based universal positive youth development program in which students in Secondary 1 to Secondary 3 participate, the Tier 2 Program is provided for students who have greater psychosocial needs at each grade. As far as the evaluation of the project is concerned, multiple evaluation strategies are used. These include objective outcome evaluation via a randomized group trial, subjective outcome evaluation based on students and program implementers, process evaluation, interim evaluation, qualitative evaluation based on students and program implementers, student products (weekly diaries and drawings), and evaluation based on personal construct psychology. Evaluation findings consistently showed that the Project P.A.T.H.S. was effective in promoting the development of adolescents joining the program [9–16].

 The constructs covered in Project P.A.T.H.S. are also covered in a university version of the program. At The Hong Kong Polytechnic University, a General Education course entitled Tomorrow's Leaders was developed to promote intrapersonal and interpersonal competencies of the students [17, 18]. Topics such as resilience, cognitive competence, social competence, emotional competence, moral competence, positive identity, and spirituality are covered in the course. Evaluation studies utilizing objective outcome evaluation, subjective outcome evaluation, and process evaluation have shown that the course was able to promote holistic development in the students taking this course [19–21].

 As there is a tendency to use positive youth development constructs in programs in high schools and universities, there is a need to review the related constructs so that program implementers and policy makers can have a better understanding of the conceptual foundations of positive youth development programs. In the conceptual review papers covered in this special issue, the following aspects are considered: definition and basic concepts of the construct, theories behind the construct, factors affecting the development of the qualities intrinsic to the construct, influence of the construct on adolescent development, and the ways to nurture the qualities related to the construct. It is our humble wish that this special issue can give a systematic account of positive youth developmental constructs so that they can be applied in the real world context.
